# Supplementary material for: Endogenous Recovery of Hippocampal Function Following Global Cerebral Ischemia in Juvenile Female Mice Is Influenced by Neuroinflammation and Circulating Sex Hormones
Source: Neural Plast. 2025 May 9;2025:6103242. doi: 10.1155/np/6103242 (PMC12084789; doi:10.1155/np/6103242)

Supplemental Figure 1: Hippocampal LTP relies on a post-synaptic mechanism. Hippocampal LTP experiments performed in Sham (A) and CA/CPR (D) animals 30 days after surgery with or without AP5. Quantification of last 10 minutes of LTP in sham (B) and CA/CPR (E) animals. PPR was unaffected by AP-5 treatment in sham (C) or CA/CPR (F) animals.


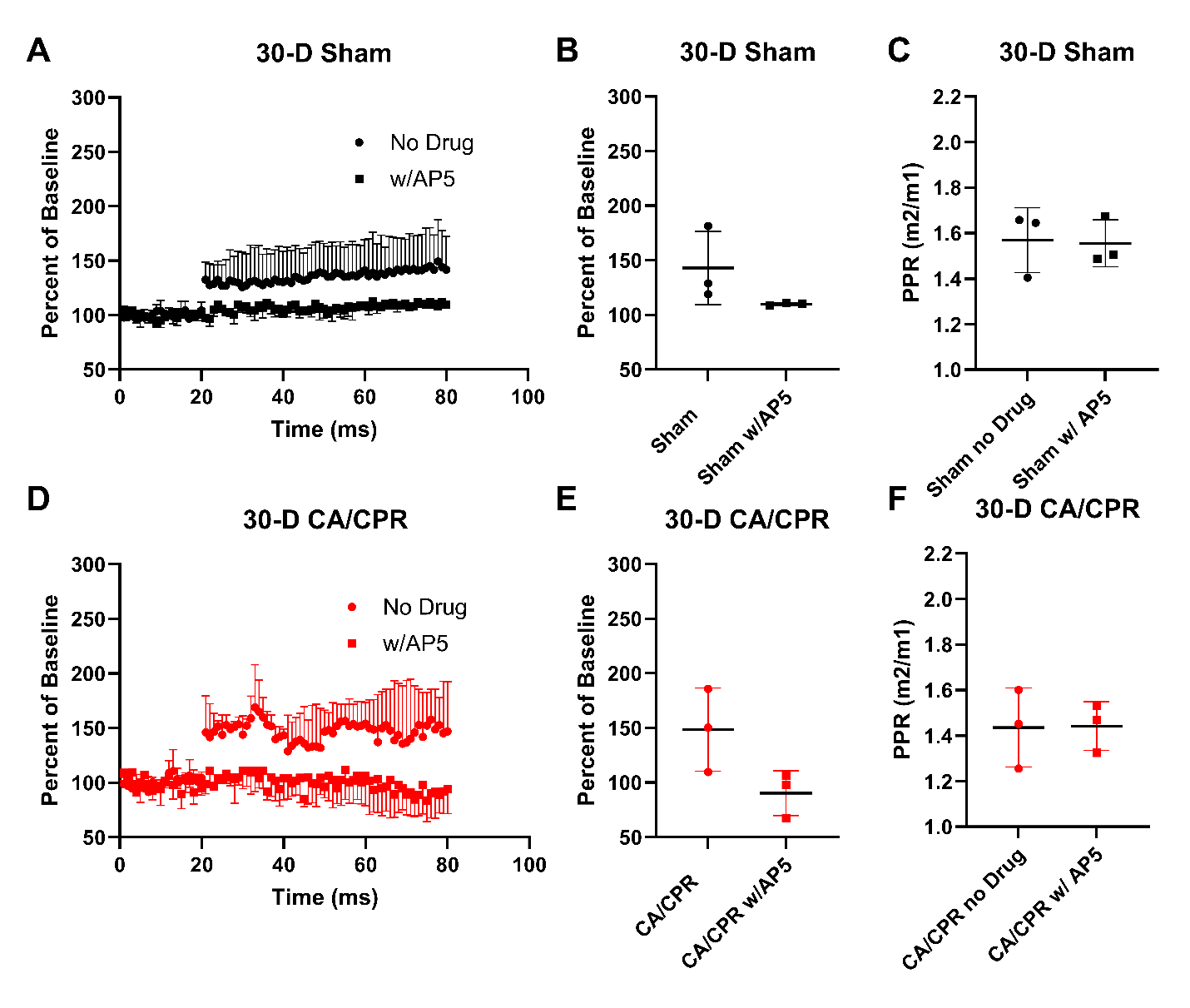

Supplement: Supporting Information — Additional supporting information for NMDA receptor dependence of LTP can be found online in the Supporting Information section. [file 6103242.f1.docx]
